# CCDC134 as a Prognostic-Related Biomarker in Breast Cancer Correlating With Immune Infiltrates

**DOI:** 10.3389/fonc.2022.858487

**Published:** 2022-03-03

**Authors:** Zhijian Huang, Linhui Yang, Jian Chen, Shixiong Li, Jing Huang, Yijie Chen, Jingbo Liu, Hongyan Wang, Hui Yu

**Affiliations:** ^1^ Department of Breast Surgical Oncology, Fujian Medical University Cancer Hospital, Fujian Cancer Hospital, Fuzhou, China; ^2^ The Graduate School of Fujian Medical University, Fuzhou, China; ^3^ Department of Pharmacy, Fujian Medical University Cancer Hospital, Fujian Cancer Hospital, Fuzhou, China; ^4^ Department of Ultrasound, Fujian Medical University Cancer Hospital, Fujian Cancer Hospital, Fuzhou, China; ^5^ Pathology Department, Daqing Longnan Hospital, The Fifth Affiliated Hospital of Qiqihar Medical College, Daqing, China; ^6^ Department of Pathology, Daqing Oilfield General Hospital, Daqing, China

**Keywords:** breast cancer, CCDC134, biomarker, prognosis, immune infiltration

## Abstract

**Background:**

The expression of Coiled-Coil Domain Containing 134(CCDC134) is up-regulated in different pan-cancer species. However, its prognostic value and correlation with immune infiltration in breast cancer are unclear. Therefore, we evaluated the prognostic role of CCDC134 in breast cancer and its correlation with immune invasion.

**Methods:**

We downloaded the transcription profile of CCDC134 between breast cancer and normal tissues from the Cancer Genome Atlas (TCGA). CCDC134 protein expression was assessed by the Clinical Proteomic Cancer Analysis Consortium (CPTAC) and the Human Protein Atlas. Gene set enrichment analysis (GSEA) was also used for pathway analysis. Receiver operating characteristic (ROC) curve was used to differentiate breast cancer from adjacent normal tissues. Kaplan-Meier method was used to evaluate the effect of CCDC134 on survival rate. The protein-protein interaction (PPI) network is built from STRING. Function expansion analysis is performed using the ClusterProfiler package. Through tumor Immune Estimation Resource (TIMER) and tumor Immune System Interaction database (TISIDB) to determine the relationship between CCDC134 expression level and immune infiltration. CTD database is used to predict drugs that inhibit CCDC134 and PubChem database is used to determine the molecular structure of identified drugs.

**Results:**

The expression of CCDC134 in breast cancer tissues was significantly higher than that of CCDC134 mRNA expression in adjacent normal tissues. ROC curve analysis showed that the AUC value of CCDC134 was 0.663. Kaplan-meier survival analysis showed that patients with high CCDC134 had a lower prognosis (57.27 months vs 36.96 months, P = 2.0E-6). Correlation analysis showed that CCDC134 mRNA expression was associated with tumor purity immune invasion. In addition, CTD database analysis identified abrine, Benzo **(**A) Pyrene, bisphenol A, Soman, Sunitinib, Tetrachloroethylene, Valproic Acid as seven targeted therapy drugs that may be effective treatments for seven targeted therapeutics. It may be an effective treatment for inhibiting CCDC134.

**Conclusion:**

In breast cancer, upregulated CCDC134 is significantly associated with lower survival and immune infiltrates invasion. Our study suggests that CCDC134 can serve as a biomarker of poor prognosis and a potential immunotherapy target in breast cancer. Seven drugs with significant potential to inhibit CCDC134 were identified.

## Introduction

Breast cancer (BC) has overtaken lung cancer as the most common cancer worldwide and is the most common cancer in women (1). Over the past 20 years, the number of new cases of breast cancer has gradually increased globally, from 1.15 million (2002) (2) to 1.38 million (2008) (3), 1.68 million (2012) (4) and 2.09 million (2018) (5); The projected figure for 2050 is about 3.2 million (6). Thanks to advances in early screening and the development of anti-cancer strategies, the treatment of breast cancer has improved significantly. However, the recurrence rate remains high (7–9). Studies have shown that the prognosis of breast cancer is influenced by a variety of clinical factors (10), such as age, tumor size, histological grade, lymphatic infiltration, number of lymph node metastases, hormone receptor status, Her-2 status, and positive margins. Due to the complexity of the onset of breast cancer and the heterogeneity of tumors, although many prognostic markers have been found, the prediction efficiency is still inadequate (11, 12). It is necessary to build a new breast cancer risk prediction model to improve the treatment and prognosis of breast cancer patients.

Relevant studies have reported that CCDCl34 is a newly discovered secreted protein screened by Huang et al. (13) through cell chip in 2008, which is widely expressed in a variety of human normal tissues, cancer tissues and cell lines. CCDC134 is a protein coding gene. Disorders associated with *CCDC134* include Ehlers-Danlos syndrome, hyperactivity type. *CCDC134* is reported to be a novel CD8+T cell stimulator that promotes proliferation and activation of CD8+ T cells in exocrine form, suggesting a cytokine like function (14). It has been reported that this gene may affect ERK and JNK signaling activity in gastric cancer cells (13, 15).

At present, the relationship between CCDCl34 and breast tumors has not been reported. We hypothesized that CCDC134 levels were associated with breast cancer survival. To test this hypothesis, we evaluated the prognostic role of CCDC134 in breast Cancer based on data from the Cancer Genome Atlas (TCGA). In this study, we found that CCDC134 is up-regulated in breast cancer. Notably, upregulation of CCDC134 was associated with adverse clinical features and risk factors. We further evaluated the diagnostic and prognostic value of CCDC134 in breast cancer and its correlation. Our study suggests that overexpression of CCDC134 in breast cancer is associated with lower survival.

## Materials and Methods

### TCGA Datasets

Download transcription and expression data of CCDC134 and corresponding clinical information from TCGA official website (16). More than 5 samples of the 33 registered cancers were selected for analysis. Finally, the RNA-SEQ gene expression data of workflow type FPKM were transformed into TPM format and log2 transformation for further study. As all data were downloaded from TCGA, no approval from the Ethics Committee was required for this study. We provided the database links for this article in **Supplementary Table 1**.

### RNA Sequencing Data of CCDC134 in Breast Cancer

RNA-seq expression data of CCDC134 in breast cancer were also downloaded from TCGA and the XIANTAO platform (https://www.xiantao.love/). Therefore, data on 1109 breast cancers and 113 adjacent normal tissues were retained. The selected samples contained CCDC134 gene expression data and relevant clinical information, including age, sex, smoking status, T stage, N stage, M stage, tumor site, ER/PR/HER2 status, etc. The mRNA expression data were the mean of X ± SD.

### Clinical Proteomic Tumor Analysis Consortium (CPTAC) and UALCAN

CPTAC (https://proteomics.cancer.gov/programs/cptac) application of proteomics techniques, by mass spectrometry analysis of tumor biological specimens, quantification and identification of each tumor sample, the composition of the protein and the protein group were characterized (17). UALCAN[http://ualcan.path.uab.edu/] is a user-friendly online resource for analyzing publicly available cancer data (18). In this study, we used UALCAN to analyze the expression of CCDC134 protein in CPTAC.

### The Human Protein Atlas (HPA)

HPA (https://proteinatlas.org/) contains normal tissue and tumor tissue protein levels of human gene expression profile information (19). In this study, we compared the expression of CCDC134 protein in normal lung tissue and breast cancer tissue by HPA.

### GSEA (Gene Set Enrichment Analysis) Functional and Pathway Analysis

GSEA (20) was performed using clusterProfiler, enrichPlot and ggplot2 R packet (V 3.3.3) to demonstrate important functions and pathways between the two groups. The expression level of CCDC134 was used as a phenotypic marker. Adjusted p values<0.05, the enrichment of standardized scores (|NES|) < 1, the false discovery rate (FDR)<0.25 is significant difference.

### Construction and Evaluation of Line Graph

Individual prediction of 1-year, 3-year and 5-year survival probability (21). Based on the results of multiple variable analysis, the column chart was constructed. The RMS R package (version 6.2-0) is used to generate a Nomogram with significant clinical features and calibration diagrams. C concordiindex and correction curve were used to estimate its predictive ability.

### Protein-Protein Interaction (PPI) Networks and Functional Enrichment Analysis

STRING is an online database for retrieving interacting genes [version 11.0 (https://www.string-db.org/)] (22). In this study, we used STRING to search for co-expressed genes and construct a PPI network (23, 24), with an interaction score of 0.4. Analysis of Kyoto Encyclopedia of Genes and Genomes (KEGG) pathways enriched by gene ontology (GO) and co-expressed genes was performed using ClusterProfiler software package and GGploT2 software package visualization (20).

### Tumor Immune Estimation Resource (TIMER) Database

The TIMER (https://cistrome.shinyapps.io/timer/) is a comprehensive online database, analysis of a wide variety of cancer types related to immune infiltrating (25). In this study, we used TIMER to determine the relationship between CCDC134 expression and six types of immune infiltrates (B cells, CD4+ T cells, CD8+ T cells, neutrophils, macrophages, and dendritic cells) in breast cancer.

### Tumor-Immune System Interaction Database (TISIDB)

TISIDB (http://cis.hku.hk/TISIDB/) is a tumor-immune system interaction online portal (26). In this study, we used TISIDB to determine CCDC134 and tumor-infiltrating lymphocyte (TILs) expression in human cancers. The relative abundance of TILs was deduced from gene expression profile and gene set variation analysis. Spearman test was used to measure the correlation between CCDC134 and TILs. The relative abundance threshold was set as |R| >0.5, P-value <0.05.

### Recurrence - Free Survival (RFS) Data Analysis

Survival data from KMPLOT online analysis database (https://kmplot.com/analysis/index.php?p = service). Affy IDS of CCDC134 are 220077_at. “Auto select best truncation value” and “Basic type” were selected for breast cancer analysis.

### Screening of Small Molecule Therapeutic Drugs

The selected genes are used for potential drug prediction in CTDBase. CTD database (https://ctdbase.org/). can be used to advance the understanding of chemical medicines and human health based on studies of the relationships between chemistry, genes, phenotypes, diseases and the environment. PubChem database (https://pubchem.ncbi.nlm.nih.gov/) is used to determine the identified molecular structure of the drug.

### Statistical Analyses

All statistical analyses were performed using R (V 3.6.3), and differences were visualized using R package GGplot2 (20), clusterProfiler package [version 3.14.3] (for GSEA analysis). Paired T test and Mann-Whitney U test were used to determine the difference between breast cancer tissue and adjacent normal tissue. ROC curves were used to detect CCDC134 cutoff values (27–29) using pROC packages.

## Results

### Expression Pattern of CCDC134 in Pan-Cancer Perspective

**Figure 1:**
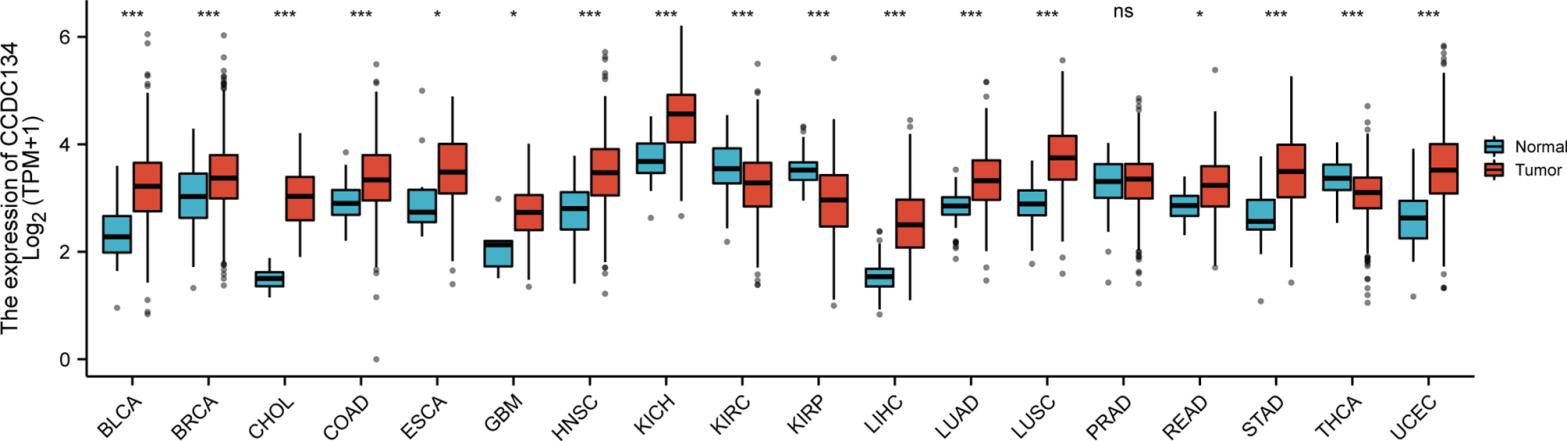
Expression pattern of CCDC134 from the perspective of pan-cancer. CCDC134 mRNA expression was significant in 17 of the 18 cancers compared with normal tissue. (ns, p≥0.05; *p< 0.05; ***p<0.001). BLCA, Bladder Urothelial Carcinoma; BRCA, Bladder Urothelial Carcinoma; CHOL, Cholangiocarcinoma, COAD, Colon adenocarcinoma; ESCA, Esophageal carcinoma, GBM, Glioblastoma multiforme; HNSC, Head and Neck squamous cell carcinoma; KICH, Kidney Chromophobe; KIRC, Kidney renal clear cell carcinoma; KIRP, Kidney renal papillary cell carcinoma; LIHC, Liver hepatocellular carcinoma; LUAD, Lung adenocarcinoma; LUSC, Lung squamous cell carcinoma; PRAD, Prostate adenocarcinoma; READ, Rectum adenocarcinoma; STAD, Stomach adenocarcinoma; THCA, Thyroid carcinoma; UCEC, Uterine Corpus Endometrial Carcinoma.

To assess the mRNA expression pattern of CCDC134 in different cancer types, 15 cancer types with fewer than 5 samples from the normal group were excluded from the analysis. The final working set involved 18 cancer types. As shown in **Figure 1**, CCDC134 was significantly upregulated in 14 of the 18 cancers compared with normal tissue. This data suggests that CCDC134 mRNA expression is abnormal in different cancer types.

### Upregulated mRNA and Protein Expression of CCDC134 in Patients With Breast Cancer

**Figure 2:**
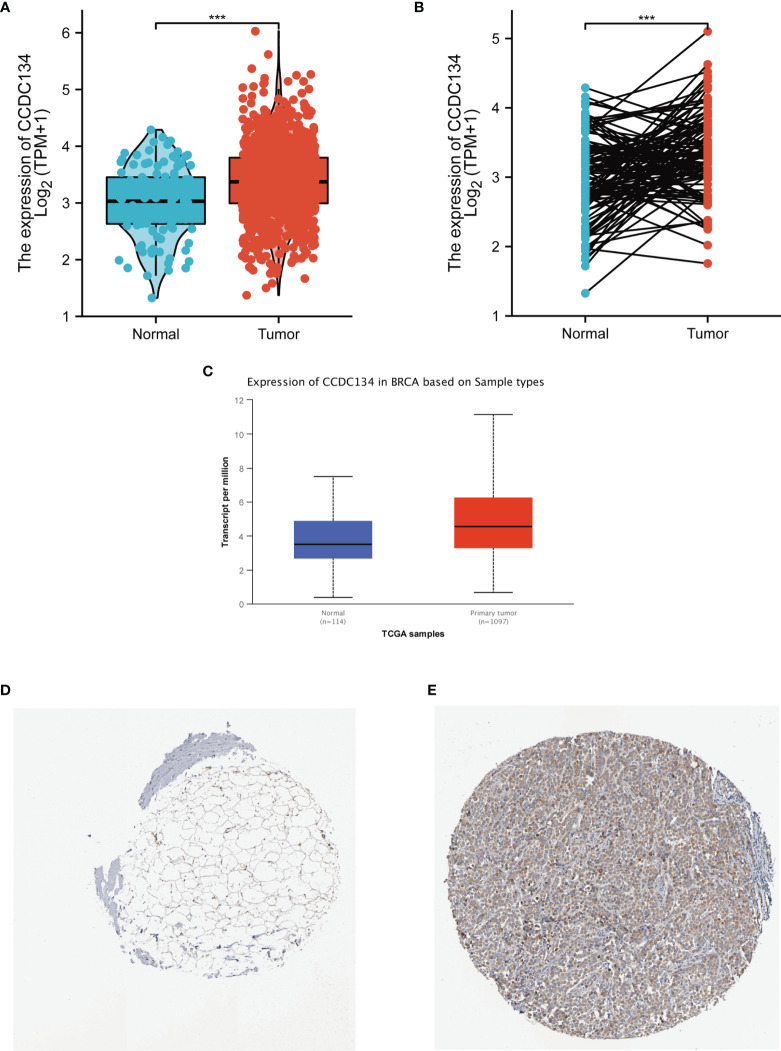
CCDC134 mRNA and protein expression in breast cancer. **(A)** CCDC134 mRNA expression levels in 1109 breast and 113 normal breast cancers. **(B)** CCDC134 mRNA expression levels in 112 breast cancer patients and matched adjacent normal samples. **(C)** CCDC134 protein expression level based on CPTAC. **(D)** CCDC134 protein levels based on Human Protein Atlas. Normal tissue, https://www.proteinatlas.org/ENSG00000100147-CCDC134/tissue/breast#img; **(E)** Tumor tissue, https://www.proteinatlas.org/ENSG00000100147-CCDC134/pathology/breast+cancer#img. (***p<0.001).

To detect CCDC134 mRNA and protein expression in breast cancer, we analyzed CCDC134 expression data in TCGA and HPA.As shown in **Figure 2A**, unpaired data analysis showed that CCDC134 mRNA expression level in breast cancer tissues (n = 1109) was significantly higher than that in normal tissues (n = 113)(**Figure 2A**, 3.391±0.636 vs 3.005±0.618, The Mann - Whitney U - test, P < 0.001). Paired data analysis also showed that mrna expression level of CCDC134 in breast cancer tissues (n = 112) was significantly higher than that in adjacent normal tissues (n = 112) (**Figure 2B**, 3.351±0.597 vs 2.998±0.617, P <0.001). In order to comprehensively analyze CCDC134 protein expression, we used UALCAN to analyze CPTAC. The results showed that CCDC134 protein expression in breast cancer was significantly higher than that in normal tissues (**Figure 2C**). As shown in **Figures 2D, E**, HPA immunohistochemical staining also showed up-regulation of CCDC134 protein expression in breast cancer tissues. These results showed that both mRNA and protein expression of CCDC134 were up-regulated in breast cancer tissues.

### Gene Set Enrichment Analysis (GSEA)

**Figure 3:**
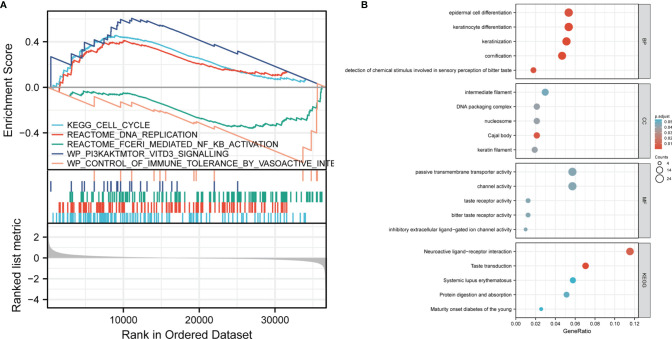
GSEA analysis results. **(A)** Genes enriched in representative pathways were analyzed by GSEA function. **(B)** GO and KEGG analysis of DEGs in low and high expression samples of CCDC134.

In order to understand the biological function of CCDC134, we analyzed the DEGs between the low and high CCDC134 expression groups according to the median expression value of CCDC134. GSEA pathway analysis was also performed (**Supplementary Table 2**) (**Figure 3**). The result shows that CCDC134 is enriched in CELL_CYCLE, REACTOME_DNA_REPLICATION, and WP_PI3KAKTMTOR_VITD3. Low tolerance_by_vasoactive_INte and REACTOME_FCERI_MEDIATED_NF_KB_ACTIVATION were found (**Figure 3**). At the same time, we completed the GO analysis(**Supplementary Table 3**). BP and keratinization, differentiation, chemical stimulation involved in detecting bitter sensory perception, epidermal cell differentiation. The first five CC terms are associated with cajal bodies, nucleosomes, DNA packaging complexes, keratin filaments, and intermediate filaments. The first five MF terms were associated with bitter taste receptor activity, channel activity, passive transmembrane transporter activity, inhibitory extracellular ligand gated ion channel activity, and taste receptor activity (**Figure 3B**).

### Clinicopathological Features

**Figure 4:**
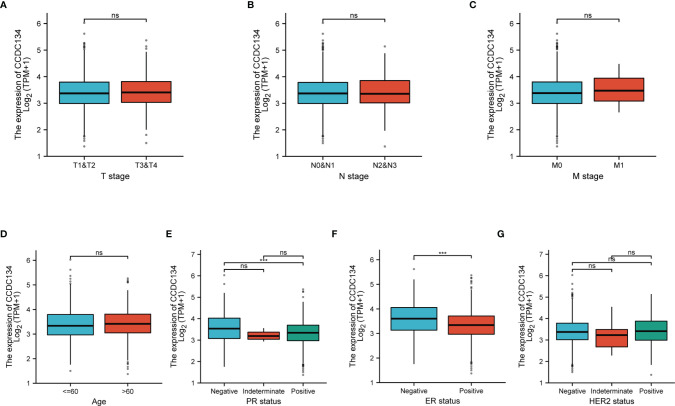
CCDC134 mRNA level relationship with clinical pathological characteristics. There was no significant difference between CCDC134 mRNA expression and T **(A)**, N **(B)**, M **(C)** level, age **(D)** and HER2 **(G)** level. It was negatively correlated with PR **(E)** and ER **(F)** status. (ns, p≥0.05; ***p<0.001).

To evaluate the relationship between CCDC134 mRNA expression and clinicopathological features in breast cancer samples, mann-Whitney U test and Logistic regression analysis were performed. As shown in **Table 1** and **Figure 4**, CCDC134 expression was observed to have a strong association with PR status (P = 0.002), ER status (P < 0.001) and PAM50 types (P < 0.001). However, CCDC134 expression was not associated with T stage (P = 0.640), N stage (P =0.545), M stage (P = 0.921), age (P = 174 0.081), pathological stage (P =0.786) and HER2 status (P =0.737). In summary, these results suggest that CCDC134 was involved in hormone receptor levels. Since the efficacy and prognosis of endocrine therapy for breast cancer were closely related to hormone receptor expression level, the results indicated that CCDC134 might be a biomarker of efficacy and prognosis of BC.

**Table 1:** Clinical characteristics of the Breast invasive carcinoma patients (TCGA).

Characteristic	Low expression of CCDC134	High expression of CCDC134	p
n	541	542	
T stage, n (%)			0.640
T1	137 (12.7%)	140 (13%)	
T2	318 (29.4%)	311 (28.8%)	
T3	72 (6.7%)	67 (6.2%)	
T4	14 (1.3%)	21 (1.9%)	
N stage, n (%)			0.545
N0	248 (23.3%)	266 (25%)	
N1	190 (17.9%)	168 (15.8%)	
N2	58 (5.5%)	58 (5.5%)	
N3	40 (3.8%)	36 (3.4%)	
M stage, n (%)			0.516
M0	450 (48.8%)	452 (49%)	
M1	8 (0.9%)	12 (1.3%)	
Age, n (%)			0.081
<=60	315 (29.1%)	286 (26.4%)	
>60	226 (20.9%)	256 (23.6%)	
Pathologic stage, n (%)			0.786
Stage I	90 (8.5%)	91 (8.6%)	
Stage II	315 (29.7%)	304 (28.7%)	
Stage III	123 (11.6%)	119 (11.2%)	
Stage IV	7 (0.7%)	11 (1%)	
Histological type, n (%)			0.001
Infiltrating Ductal Carcinoma	373 (38.2%)	399 (40.8%)	
Infiltrating Lobular Carcinoma	126 (12.9%)	79 (8.1%)	
PR status, n (%)			0.002
Negative	145 (14%)	197 (19.1%)	
Indeterminate	3 (0.3%)	1 (0.1%)	
Positive	365 (35.3%)	323 (31.2%)	
ER status, n (%)			<0.001
Negative	91 (8.8%)	149 (14.4%)	
Indeterminate	1 (0.1%)	1 (0.1%)	
Positive	421 (40.7%)	372 (35.9%)	
HER2 status, n (%)			0.737
Negative	279 (38.4%)	279 (38.4%)	
Indeterminate	7 (1%)	5 (0.7%)	
Positive	75 (10.3%)	82 (11.3%)	
PAM50, n (%)			<0.001
Normal	22 (2%)	18 (1.7%)	
LumA	310 (28.6%)	252 (23.3%)	
LumB	103 (9.5%)	101 (9.3%)	
Her2	35 (3.2%)	47 (4.3%)	
Basal	71 (6.6%)	124 (11.4%)	

### Diagnostic and Prognostic Value

**Figure 5:**
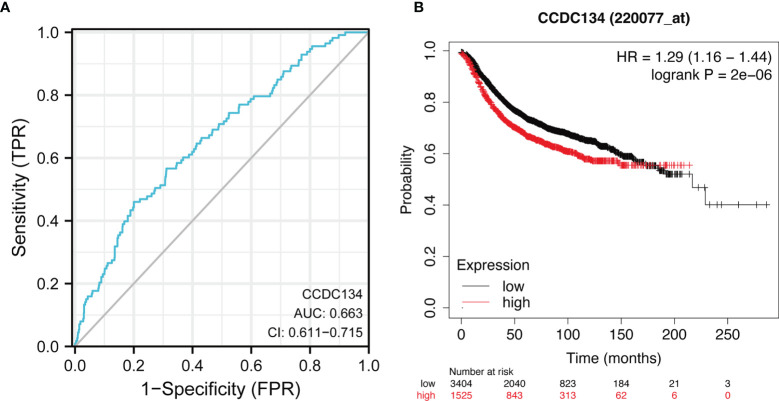
ROC and Kaplan-Meier curves of CCDC134. **(A)** THE ROC curve showed that the AUC value of CCDC134 was 0.663. **(B)** Kaplan-Meier survival curve showed that the RFS of breast cancer patients with high CCDC134 mRNA expression was shorter (57.27 months vs. 36.96 months) than that of breast cancer patients with low CCDC134 mRNA expression. P = 2.0 e-6).

In order to study the diagnostic value of CCDC134 in distinguishing breast cancer samples from normal breast cancer, ROC curve analysis was conducted. As shown in **Figure 5A**, ROC curve analysis shows that the AUC value of CCDC134 is 0.663, 95% Confidence interval (95% CI) = 0.611-0.715. When the critical value was 2.904, the sensitivity was 0.460 and the specificity was 0.800. The positive predictive value was 0.190, and the negative predictive value was 0.936. The results suggest that CCDC134 may be a promising biomarker for differentiating adenocarcinoma tissue from normal tissue. The relationship between CCDC134 mRNA expression and RFS in breast cancer patients was explored by Kaplan-Meier curve. As shown in **Figure 5B**, the RFS of patients with high level CCDC134 breast cancer was shorter than that of patients with low level CCDC134 breast cancer (57.27 months vs. 36.96 months, P = 2.0E-6).

### Constructed a Nomogram Diagram

**Figure 6:**
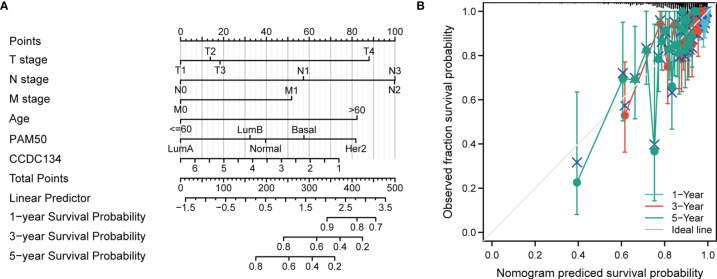
Construction of a rosette to predict the probability of survival in BRCA patients. **(A)** Nomogram to predict 1 -, 3 -, and 5-year BRCA survival probabilities consisting of CCDC134 and independent clinical risk factors. **(B)** Nomograms calibrated to predict the probabilities of 1 -, 3 - and 5-year survival. The gray line represents actual survival.

To provide a quantitative method for predicting the prognosis of BRCA patients, we constructed a Nomogram diagram of CCDC134 and independent clinical risk factors (T/N/M stage, CCDC134, age, PAM50). In this Nomogram based on multivariate Cox analysis, a point scale is used to assign points to these variables. Draw a straight line up to determine the number of points for a variable and adjust the sum of points assigned to each variable to a range from 0 to 100. The integrals of the variables are added up and recorded as an overall score. The 1-year, 3-year, and 5-year survival probabilities for BRCA patients were determined vertically from the total point axis down to the outcome axis (**Figure 6A**). The nomogram C-index of OS is predicted to be 0.729 (0.705-0.753) (**Figure 6B**).

### PPI Network and Functional Annotation

**Figure 7:**
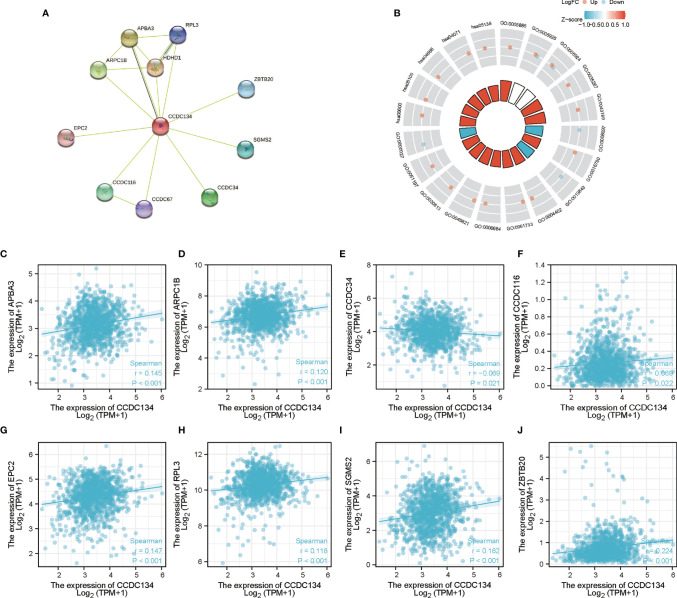
PPI network and functional enrichment analysis. **(A)** CCDC134 and its co-expressed gene network. **(B)** Functional enrichment analysis of co-expressed genes. **(C–J)** The correlation analysis of CCDC134 expression and co-expressed genes.

To build the PPI network and functional annotations, we performed STRING database, GO and KEGG analysis. **Figure 7A** showed the network of CCDC134 and its 10 co-expressed genes (**Supplementary Table 4**). As shown in **Figure 7B**, changes in CCDC134 bioprocesses were related to sphingomyelin metabolism, active regulation of glycolysis, positive regulation of nucleotide catabolism, active regulation of coenzyme metabolism, and assembly of ribosome macrosubunits. Functional annotations showed that these genes were involved in 5S rRNA binding, phosphotransferase activity for other substituted phosphate groups, rRNA binding, histone acetyl transferase activity, peptide-lysine-N-acetyl transferase activity and other functions. The correlation analysis of CCDC134 expression and co-expressed genes in TCGA breast cancer was shown in **Figures 7C–J** (successfully converted to 9 Entrez ID).

### Correlation Between CCDC134 and Immune Cell Infiltration

**Figure 8:**
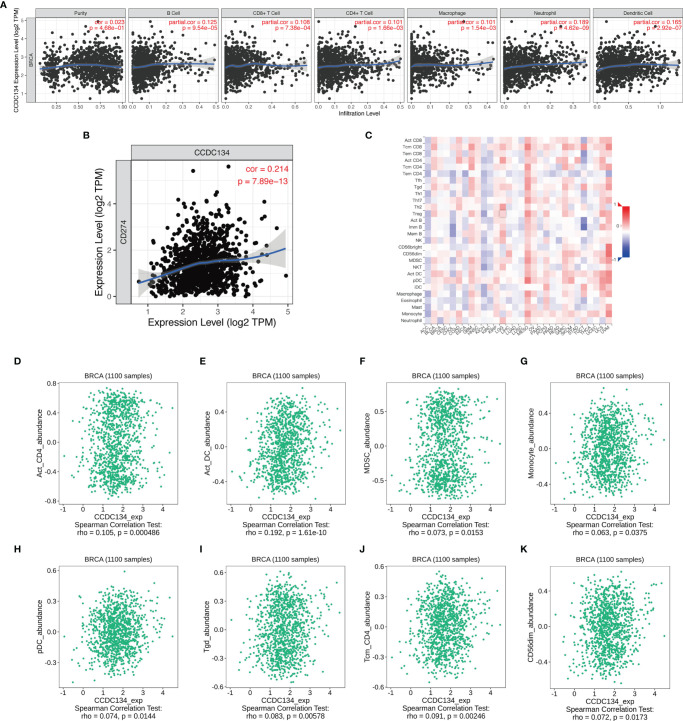
CCDC134 expression and the immune level of correlation. **(A)** The expression of CCDC134 in breast cancer was correlated with tumor purity, B cells, CD4 + T cells, CD8 + T cells, macrophages, neutrophils and dendritic cells. **(C)** Relationship between CCDC134 expression and 28 lymphocyte species in human tumors. **(B)** CCDC134 expression was positively correlated with CD274(PD-L1) in breast cancer. **(D–K)** CCDC134 and CD4 + T cells (r= 0.105, P = 0.000486), DC cells (r= 0.192, P = 1.61E-10), MDSC cells (r= 0.073, P = 0.0153), monocytes (r = 0.063, P = 0.0375), pDC cells (r = 0.074, P = 0.0144), Tgd cells (r = 0.083, P = 0.00578), Tcm_CD4 + T cells (r= 0.091, P = 0.00246) and CD56DIM (r= 0.072, P = 0.0173).

We analyzed the correlation between CCDC134 expression and six types of tumor-infiltrating immune cells in the TIMER database. As shown in **Figure 8A**, CCDC134 expression was correlated with tumor purity (r = 0.023, P = 4.68E −01), B cells (R = 0.125, P = 9.54E −05), CD8+ T cells (r =0.108, P = 7.38E −04), CD4+ T cells (r = 0.101, 1.66E-03), macrophages (r = 0.101, P = 1.54E−03), neutrophils (r = 0.189, P = 4.62E−09) and dendritic cells (r = 0.165, P = 2.92E−07) were correlated. **Figure 8B** shows the correlation between CCDC134 and PD-L1 (CD274) (r =0.214, P = 7.89E−13). We also assessed the correlation between CCDC134 expression and 28 types of lymphocytes in the TISIDB database. **Figure 8C** shows the relationship between CCDC134 expression and 28 types of lymphocytes in human cancers. As shown in **Figures 8D–K**, the expression of CCDC134 was correlated with that of CCDC134 and Act_CD4 + T cells (r= 0.105, P = 0.000486), DC cells (r= 0.192, P = 1.61E-10), MDSC cells (r= 0.073, P = 0.0153), monocytes (r = 0.063, P = 0.0375), pDC cells (r = 0.074, P = 0.0144), Tgd cells (r = 0.083, P = 0.00578) and the abundance of Tcm_CD4 + T cells (r= 0.091, P = 0.00246) and CD56DIM (r= 0.072, P = 0.0173). These data suggest that CCDC134 may play a specific role in immune invasion of breast cancer.

### Small Molecule Therapeutics

**Figure 9:**
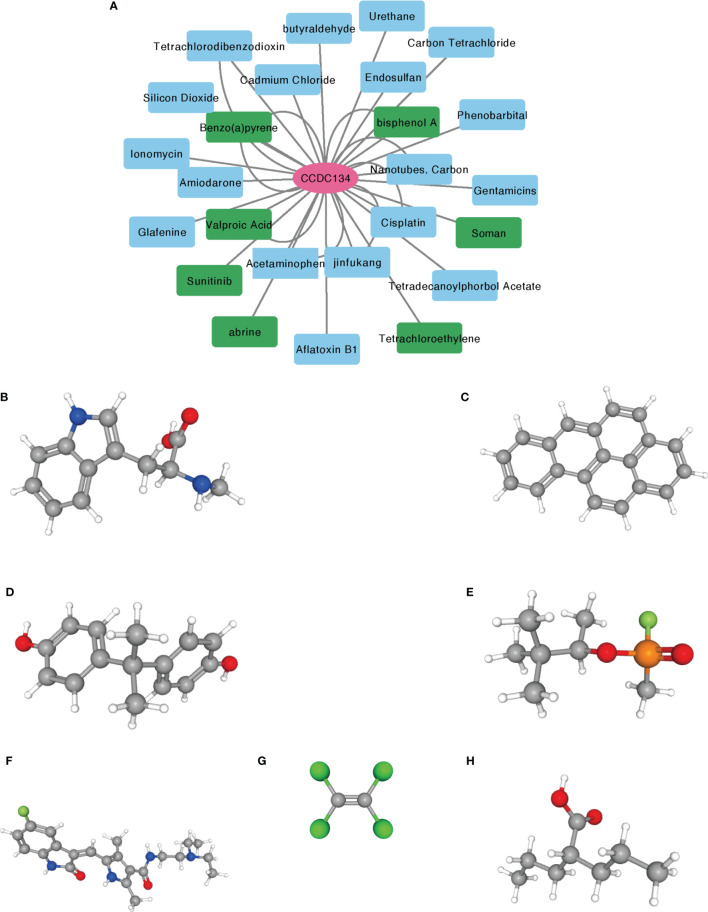
Prediction of potential drug and molecular structure affecting CCDC134. **(A)** CTD database predicts potential drugs that affect CCDC134, with green representing drug molecules that inhibit CCDC134. **(B–H)** PubChem database predicts the molecular structures of seven targeted drugs. **(B)** abrine, **(C)** Benzo(A) Pyrene, **(D)** bisphenol A, **(E)** Soman, **(F)** Sunitinib, **(G)** Tetrachloroethylene, **(H)** Valproic Acid.

The correlation between CCDC134 and potential drugs was analyzed using CTD database(**Supplementary Table 5**). A total of seven drugs were identified, including abrine, Benzo(A) Pyrene, bisphenol A, Soman, Sunitinib, Tetrachloroethylene and Valproic Acid (**Figure 9**). These drugs have a potential inhibitory effect on CCDC134.

## Discussion

In this study, we found that CCDC134 mRNA and protein expression were up-regulated in breast cancer tissues. ROC curve analysis indicated that CCDC134 may be a promising diagnostic biomarker for differentiating breast cancer from normal tissues. Using Kaplan-Meier curves and univariate analysis, we confirmed that CCDC134 expression is associated with short RFS and that CCDC134 can serve as a potential biomarker of poor prognosis in breast cancer. In addition, CCDC134 may play a specific role in immune invasion of breast cancer.

Studies have shown (30) that CCDC134 is a novel gene involved in severe progressive deformation recessive osteogenesis imperfecta (type III). (15)Decreased CCDC134 expression has been reported to enhance erK1/2 activation and JNK/SAPK expression. CCDC134 regulates cell migration and invasion and may be a therapeutic target for gastric cancer. Exposure to CCDC134 promotes proliferation of CD8 + T cells through the Janus kinase 3- signal transductor and transcriptional activator 5 pathway. Two members of the γ c cytokine family effectively block CCDC134 binding to activated CD8 + T cells. This provides evidence that CCDC134 may be a potential member of the γ c cytokine family (31).

Huang, J (32) showed that CCDC134 may act as a new regulator of hADA2a and play a role in the PCAF complex through hADA2a, affecting its acetyltransferase activity and UV-induced DNA damage repair. However, in breast cancer, CCDC134 expression and its prognostic value have not been fully studied. Systematic approaches to breast cancer include surgery, endocrine therapy, radiotherapy and targeted therapy, and Endocrine therapy is of great significance in the treatment of patients with hormone receptor positivity. In the estrogen receptor. Positive (ER+) patients, 5 years of adjuvant tamoxifen (that suppresses ER) reduces breast cancer mortality by approximately one third (0-14 years), recurrence about half (0-4 years) and about one third (5-9 years) (33). We found that the upregulation of CCDC134 was negatively correlated with hormones. In addition, since ER and PR status are correlated with prognosis, CCDC134 may be used as a predictor of hormone receptor status, and whether endocrine therapy biomark is needed. Furthermore, according to kaplan-Meier curves and log-rank tests, breast cancer patients with high mRNA expression had lower survival rates than those with low CCDC134 levels. Based on our data, we conclude that CCDC134 can serve as a biomarker for poor prognosis in breast cancer. To identify patients with poor clinical prognosis.

There is little research on the possible role of CCDC134 in human lymphocytes. The correlation analysis between CCDC134 expression and immune cell infiltration in breast cancer has not been studied. In this study, we used TIMER to find that this gene could cause an increase in the number of infiltrating immune cells such as CD8+T in tumor tissues, but the expression of this gene was also positively correlated with the expression of PD-L1. Therefore, although this gene could recruit immune cells into tumor tissues, it could also lead to an increase in the expression of PD-L1 on the surface of tumor cells. Therefore, the high expression of this gene still has an inhibitory effect against tumor immune response. It is necessary to further study the mechanism and develop corresponding targeted drugs to remove the immunosuppressive effect of this gene and improve the survival prognosis of tumor patients.

By using the CTD database to predict drugs that inhibit CCDC134, seven drugs were identified. Previous studies supported abrine, Benzo(A) Pyrene, bisphenol A, Soman, Sunitinib, Tetrachloroethylene, and Valproic Acid can target CCDC134 *in vitro* and is expected to make new progress in the treatment of breast cancer. PubChem databases were used to determine the molecular structure of identified drugs.

There are several limitations to this study. Firstly, the expression and prognostic significance of CCDC134 were studied through online public database. Further studies on clinical samples and cell animal experiments are needed to verify these results. Secondly, *in vitro* and *in vivo* experiments need to be designed in order to further study the detailed mechanism of CCDC134 affecting immune invasion of breast cancer. Thirdly, besides CCDC134, there might be other novels genes associated with breast cancer (34). It might be better to study the association between them. Finally, all analyses in this study focus on bulk sequencing data. Fourthly, it might be better to study the role of CCDC134 using single cell sequencing to avoid cell heterogeneity using methods like single cell clustering (35–38).

In summary, in this study, we found for the first time that CCDC134 is highly up-regulated in breast cancer, and poor prognosis can be used as a potential prognostic marker and may play a specific role in immune infiltration.

## Data Availability Statement

The original contributions presented in the study are included in the article/**Supplementary Material**. Further inquiries can be directed to the corresponding authors.

## Ethics Statement

Written informed consent was obtained from the individual(s) for the publication of any potentially identifiable images or data included in this article.

## Author Contributions

Authors HY and HW conceived and designed the study. ZH, LY, JC, and JH performed the experiments. YC, JC, SL, and JL analyzed the data. ZH wrote the manuscript. All authors have read and approved this manuscript.

## Funding

This work was supported by The Natural Science foundation of Fujian province (No. 2020J011112) and Joint Funds for the innovation of science and Technology, Fujian province (Grant number: 2020Y9039).

## Conflict of Interest

The authors declare that the research was conducted in the absence of any commercial or financial relationships that could be construed as a potential conflict of interest.

## Publisher’s Note

All claims expressed in this article are solely those of the authors and do not necessarily represent those of their affiliated organizations, or those of the publisher, the editors and the reviewers. Any product that may be evaluated in this article, or claim that may be made by its manufacturer, is not guaranteed or endorsed by the publisher.
