# Supplementary material for: CCDC134 as a Prognostic-Related Biomarker in Breast Cancer Correlating With Immune Infiltrates
Source: Front Oncol. 2022 Mar 3;12:858487. doi: 10.3389/fonc.2022.858487 (PMC8927640; doi:10.3389/fonc.2022.858487)
Supplement: Supplementary file 1 [file DataSheet_1.zip › Supplementary Table 1.docx]

The database links for this article as follow:

1. <https://portal.gdc.cancer.gov/>.

2. <https://proteinatlas.org/>.

3. <https://www.xiantao.love/>

4. <https://proteomics.cancer.gov/programs/cptac>.

5. <https://www.string-db.org/>.

6. <https://cistrome.shinyapps.io/timer/>.

7. <https://cistrome.shinyapps.io/timer/>.

8. https://www.gsea-msigdb.org/gsea/index.jsp.
